# Tissue-specific deposition, speciation and transport of antimony in rice

**DOI:** 10.1093/plphys/kiae289

**Published:** 2024-05-18

**Authors:** Hengliang Huang, Naoki Yamaji, Jian Feng Ma

**Affiliations:** Institute of Plant Science and Resources, Okayama University, Kurashiki 710-0046, Japan; Institute of Plant Science and Resources, Okayama University, Kurashiki 710-0046, Japan; Institute of Plant Science and Resources, Okayama University, Kurashiki 710-0046, Japan

## Abstract

Rice (*Oryza sativa*) as a staple food is a potential intake source of antimony (Sb), a toxic metalloid. However, how rice accumulates this element is still poorly understood. Here, we investigated tissue-specific deposition, speciation, and transport of Sb in rice. We found that Sb(III) is the preferential form of Sb uptake in rice, but most Sb accumulates in the roots, resulting in a very low root-to-shoot translocation (less than 2%). Analysis of Sb deposition with laser ablation-inductively coupled plasma-mass spectrometry showed that most Sb deposits at the root exodermis. Furthermore, we found that Sb is mainly present as Sb(III) in the root cell sap after uptake. Further characterization showed that Sb(III) uptake is mediated by Low silicon rice 1 (Lsi1), a Si permeable transporter. Lsi1 showed transport activity for Sb(III) rather than Sb(V) in yeast (*Saccharomyces cerevisiae*). Knockout of *Lsi1* resulted in a significant decrease in Sb accumulation in both roots and shoots. Sb concentration in the root cell sap of two independent *lsi1* mutants decreased to less than 3% of that in wild-type rice, indicating that Lsi1 is a major transporter for Sb(III) uptake. Knockout of *Lsi1* also enhanced rice tolerance to Sb toxicity. However, knockout of Si efflux transporter genes, including *Lsi2* and *Lsi3*, did not affect Sb accumulation. Taken together, our results showed that Sb(III) is taken up by Lsi1 localized at the root exodermis and is deposited at this cell layer due to lack of Sb efflux transporters in rice.

## Teaser

The toxic metalloid antimonite is taken up by a silicon influx transporter in rice and deposited at the root exodermis due to lack of efflux transporters, resulting in low translocation to the shoots.

## Introduction

Antimony (Sb) is a metalloid, which is present in trace amounts in natural environment. In general, its concentration in most soil is below 1 mg/kg (Boyle and Jonasson 1973; Lintschinger et al. 1998). However, in some areas, Sb concentration in soil reaches a high level mainly due to anthropogenic activities such as mining, smelting, shooting, and burning of fossil fuels (He 2007; Qi et al. 2011; He et al. 2012; Li et al. 2017). For example, Sb concentration in soil was as high as 100.6 to 5,045.0 mg/kg in different sites at an antimony mining and smelting area in Hunan, China (He 2007), where rice (*Oryza sativa*) is cultivated. Qi et al. (2011) also reported that in Xikuangshan Sb deposit, the total Sb value in 121 samples ranged from 79.63 to 54,221.71 mg/kg with an average of 5,949.20 mg/kg. Since Sb is a non-essential element for all organisms including plants and humans, its high concentration causes biological toxicity. In plants, high Sb concentration causes retarded growth, reduced biomass and photosynthesis, generation of reactive oxygen species, and lipid peroxidation (Pan et al. 2011; Feng et al. 2013, 2016; Zhou et al. 2018). For instance, Sb decreased rice yield by 10% when grown in a soil with the antimonite {Sb(III)} at 150 mg/kg (He and Yang 1999). For humans, Sb has been listed as a potential carcinogen (NTP 2018). Once soil is contaminated by Sb, it will be taken up by crops and subsequently enter the food chain, which presents a health risk for humans (Tschan et al. 2009a). Furthermore, Sb at a concentration unaffecting crop growth and productivity will also pose a health risk for humans through consuming foods produced over longer periods (Tylenda et al. 2015; Long et al. 2019; Vidya et al. 2022). Therefore, reducing the transfer of Sb from soil to the edible parts of crops is an important issue for human health.

Rice is a staple food for nearly half of the world's population; therefore, it could be a potential intake source of Sb. In fact, it was reported that rice contributes 33% of total Sb intake (Wu et al. 2011; Okkenhaug et al. 2012). However, little is known on the mechanisms underlying the Sb accumulation in rice. There are two major forms of inorganic Sb in soil solution: Sb(III) and antimonate {Sb(V)}, depending on soil condition (Kang et al. 2000; Mitsunobu et al. 2010). Under anaerobic conditions, Sb(III) is the predominant form due to the reduction under submerged soil, while Sb(V) could be the predominant one under aerobic conditions (Hockmann et al. 2014; Nakamaru and Altansuvd 2014). Rice plants are usually exposed to regimes of flooding and drought several times for optimal productivity during a single growing season although most time under submerged conditions. Therefore, both Sb(III) and Sb(V) could be present during the whole growth period. These forms are taken up by the roots as the first step entering the plant cells. It was reported that rice roots take up more Sb(III) than Sb(V) when the same concentration was supplied (Huang et al. 2012a; Ren et al. 2014). The uptake of Sb(V) was proposed to be mediated by Cl^−^ transporter (Tschan et al. 2009b), but not by phosphate {P(V)} transporter because of the different spatial structure between P(V) and Sb(V). In fact, addition of P(V) did not show a competitive effect on Sb(V) uptake (Tschan et al. 2008), although very high P(V) addition inhibited Sb(V) uptake (Feng et al. 2019). By contrast, uptake of Sb(III) was suggested to be mediated by some transporter members belonging to aquaporin family in plants. Several lines of evidence support this suggestion. Firstly, the uptake of arsenite {As(III)} was inhibited by its analog, Sb(III) in rice (Meharg and Jardine 2003), while As(III) is taken up mainly by Low silicon rice 1 (Lsi1), a silicon (Si) transporter belonging to aquaporin (Ma et al. 2006), suggesting that Sb(III) and As(III) share the same transporter. Secondly, Sb(III) uptake was inhibited by HgCl_2_, an inhibitor of aquaporin (Feng et al. 2019). Thirdly, Sb(III) uptake was reduced by addition of Si and the tolerance to Sb toxicity became higher in *lsi1* mutant (Huang et al. 2012b). However, the exact transporters for Sb(III) uptake in rice remain unclear. In the present study, we physiologically characterized Sb accumulation in rice in terms of uptake kinetics, tissue-specific deposition, partition, and speciation of Sb at realistic Sb concentrations. We further identified the transporter involved in Sb uptake in rice roots.

## Results

### Physiological characterization of Sb accumulation at realistic Sb concentrations

**Figure 1.**
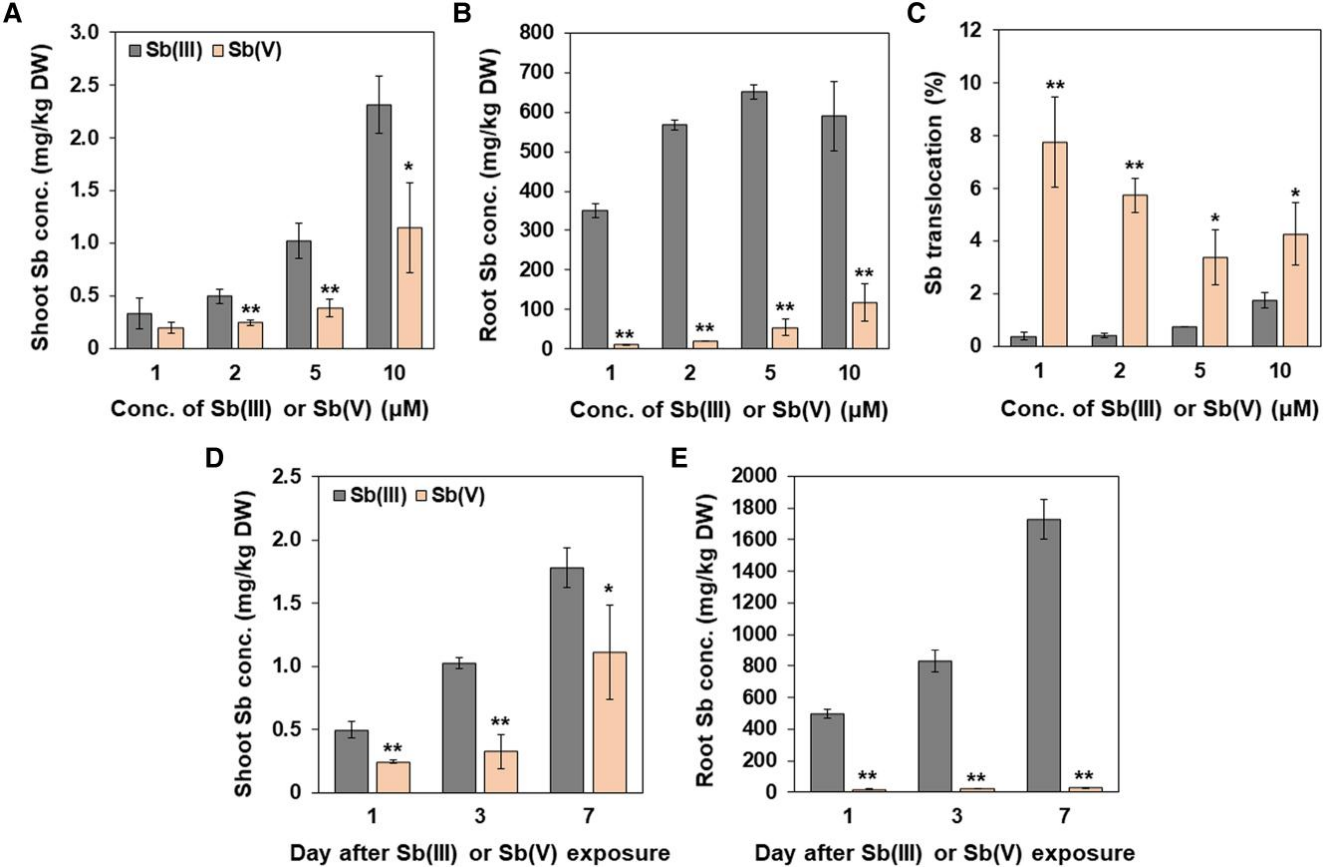
Physiological characterization of Sb accumulation in rice. **A** to **C)** Dose-dependent accumulation of Sb in the shoots A), roots B), and root-to-shoot translocation **C)**. 24-d-old seedlings of rice (cv. Nipponbare) were exposed to 1, 2, 5, or 10 *μ*M Sb(III) and Sb(V) for 1 d. **D** to **E)** Time-dependent Sb accumulation in the shoots (D) and roots (E). Rice seedlings were exposed to 2 *μ*M Sb(III) or Sb(V) for 1, 3, and 7 d. The concentration of Sb in shoots (A and D) and roots (B and E) was determined by ICP-MS after digestion. The ratio of root-to-shoot translocation (C) was calculated by Sb content in shoots/total Sb content × 100. Data are means ± Sd of three biological replicates. Significant differences between Sb(III) and Sb(V) are marked with *P < 0.05; **P < 0.01, by Student's *t*-test. Conc., concentration; DW, dry weight.

Accumulation of Sb in rice was investigated in several studies, but in most studies, high Sb concentrations were used (e.g. Huang et al. 2012a; Feng et al. 2019; Lu et al. 2023; Ran et al. 2023). For example, Feng et al. (2019) used more than 80 *μ*M Sb for their treatments. Given low solubility and mobility of Sb in soil, Sb concentration in soil solution is usually very low even in Sb-contaminated soil. For example, soil contaminated with 50 to 100 mg/kg Sb contained around 0.8 to 10 *μ*M Sb(III) in the solution (Hammel et al. 2000; Long et al. 2019). We therefore, firstly characterized the Sb accumulation at realistic Sb concentrations from 1 *μ*M to 10 *μ*M. When the rice plants (24-d-old) were exposed to Sb(III) or Sb(V) at different concentrations for one day, both the roots and shoots accumulated more Sb when Sb(III) was supplied than Sb(V) was (Fig. 1, A and B). Especially the roots exposed to Sb(III) accumulated 4 to 30 times more Sb compared with the roots exposed to Sb(V) (Fig. 1B). The Sb concentration in the shoots increased with increasing external Sb(III) or Sb(V) supplied, but the Sb accumulation in the roots was saturated when Sb(III) added was higher than 5 *μ*M, although it was slightly increased in the roots exposed to Sb(V) (Fig. 1B). The ratio of root-to-shoot translocation was lower than 2% in plants exposed to Sb(III) (Fig. 1C), while it was 8% in plant exposed to Sb(V), indicating most Sb is retained in the roots, especially in plants exposed to Sb(III).

A time-course experiment showed that Sb accumulation in both the shoots and roots increased with prolonged exposure times (Fig. 1, D and E), but at all time points, plants exposed to Sb(III) always accumulated more Sb in both the roots and shoots compared with those exposed to Sb(V).

### Partition of Sb in rice roots

**Figure 2.**
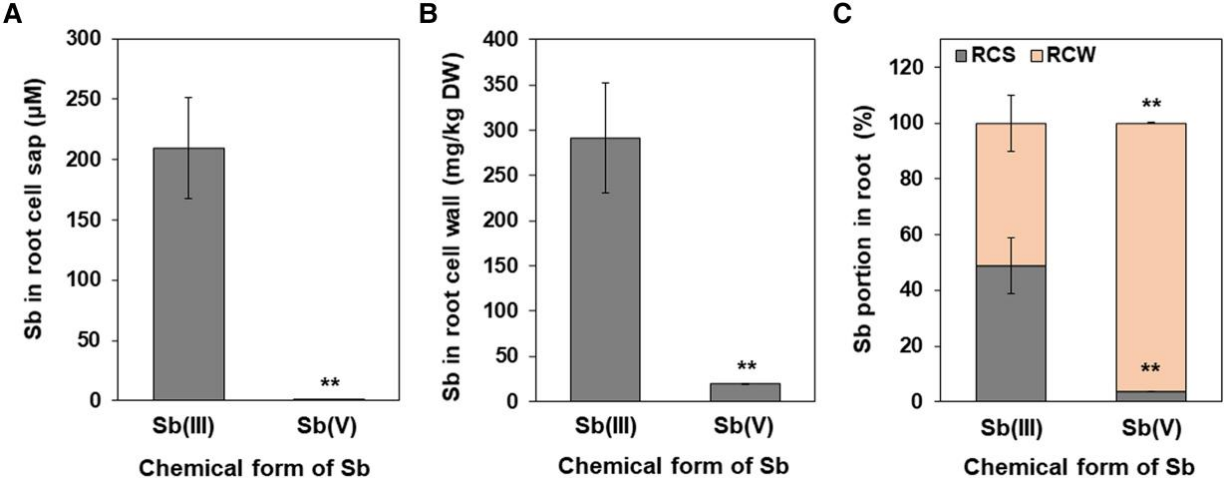
Partitioning of Sb in rice roots. **A)** Sb concentration in the root cell sap. **B)** Sb concentration in the root cell wall. **C)** Partition of Sb in the cell sap and cell wall. Rice seedlings (24-d-old, cv. Nipponbare) were exposed to 2 *μ*M Sb(III) or Sb(V). After 24 h, the root cell sap was collected by centrifugation and the remaining part was subjected to digestion after dried. The concentration of Sb in root cell sap and digestion solution was determined by ICP-MS. Sb portion in the root cell sap (RCS) and root cell wall (RCW) (C) were calculated based on content in each part. Data are means ± Sd of three biological replicates. Significant differences between Sb(III) and Sb(V) are marked with **P < 0.01, by Student's *t*-test. DW, dry weight.

Since most Sb was accumulated in the roots (Fig. 1, B and E), we examined the partition of Sb in the root cell wall and cell sap (soluble Sb within the cells). When the seedlings (24-d-old) were exposed to 2 *μ*M Sb(III) for 24 h, the Sb concentration in the root cell sap was as high as 200 *μ*M (Fig. 2A), which accounted for 49% of the total Sb in the roots. By contrast, when Sb(V) was supplied, the Sb concentration in the root cell sap was very low (0.5 *μ*M), and most Sb was bound in the cell wall (Fig. 2, B and C).

### Speciation of Sb in the root cell sap and xylem sap

**Figure 3.**
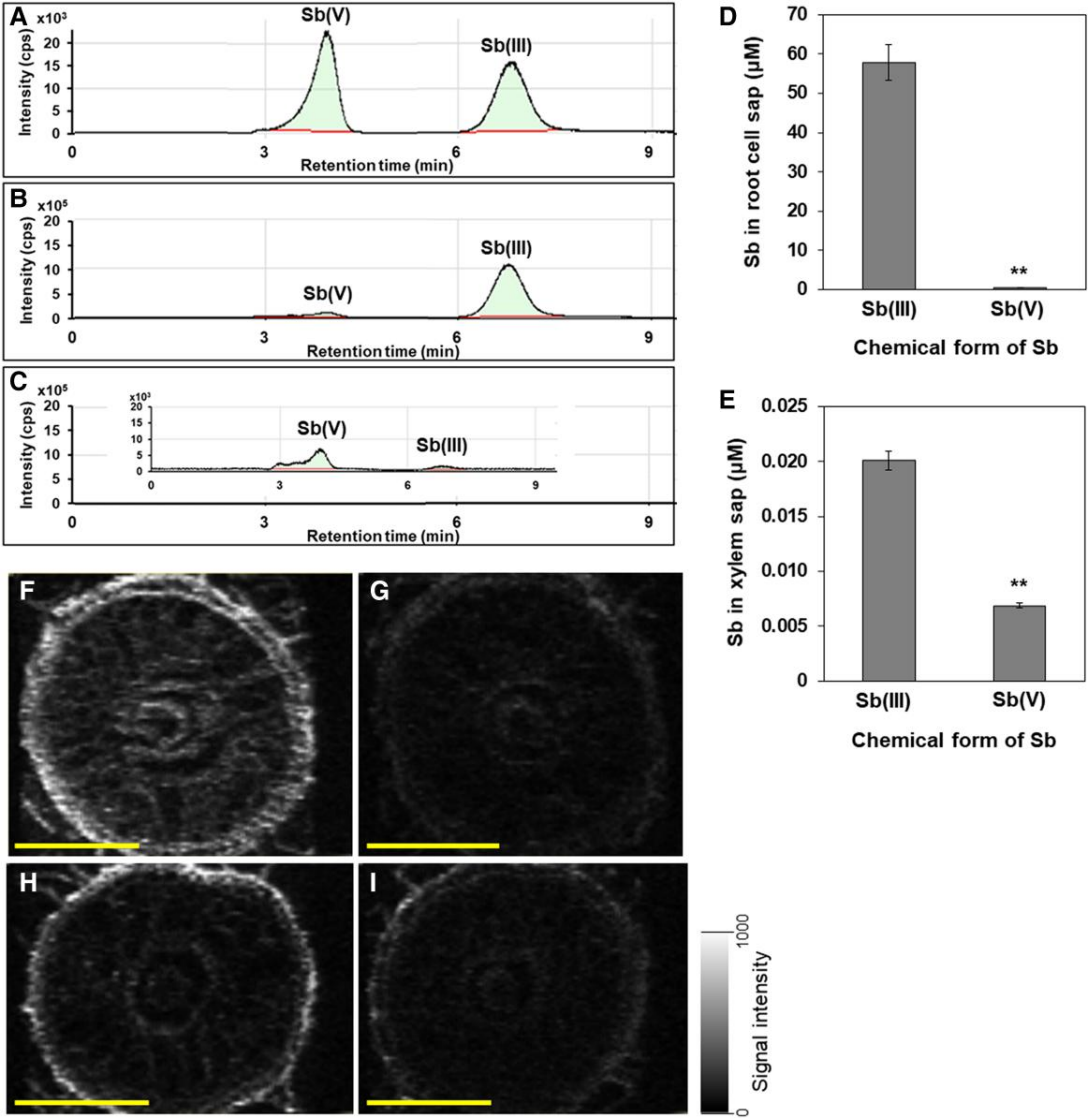
Speciation and deposition pattern of Sb in rice roots. **A** to **C)** Chromatograms of standard solution [100 ppb Sb(III) and Sb(V)] (A) and root cell sap (B-C). **D** to **E)** Concentration of Sb in root cell sap (D) and xylem sap (E). Rice seedlings (22-d-old, cv. Nipponbare) were exposed to a nutrient solution containing 2 *μ*M Sb(III) (B) or Sb(V) (C) for 4 h. The root cell sap was collected and used for Sb speciation measurement by HPLC-ICP-MS. The xylem sap was collected for Sb determination by ICP-MS. Data are means ± Sd of 3 biological replicates. Significant differences between Sb(III) and Sb(V) are marked with **P < 0.01, by Student's *t*-test. **F** to **I)** Deposition pattern of Sb in roots of both WTs [Nipponbare (F) and Oochikara (H)] and mutants [*lsi1-1* (G) and *lsi1-2* (I)]. 5-d-old seedlings were exposed to a solution containing 2 *μ*M Sb(III) for 4 h. Roots (20 mm from the root apex) of each line were sampled for Sb mapping by LA-ICP-MS. Scale bars = 200 *μ*m.

We investigated chemical forms of Sb in the root cell sap by HPLC-ICP-MS. Sb(III) and Sb(V) gave different retention time (Fig. 3A). After the seedlings (22-d-old) were exposed to 2 *μ*M Sb(III) for 4 h, the Sb in the root cell sap was present mainly in the form of Sb(III) (Fig. 3B). Similarly, when the plants were exposed to Sb(V), this form remained in the root cell sap although the peak intensity was much lower compared with that of Sb(III) (Fig. 3, B and C).

We also determined Sb concentration in the xylem sap compared with that in the root cell sap using the same plants. The concentration of Sb in the xylem sap was much lower than that in the cell sap of the roots exposed to either Sb(III) or Sb(V) (Fig. 3, D and E). For example, in contrast to 58 *μ*M Sb in the root cell sap, only 0.02 *μ*M Sb was detected in the xylem sap (Fig. 3, D and E). Plants exposed to Sb(III) showed higher Sb concentration in the xylem sap than those exposed to Sb(V). Since the Sb concentration in the xylem sap was too low, we failed to analyze its speciation accurately.

### Tissue-specific accumulation of Sb in roots

With help of LA-ICP-MS, we mapped the deposition of Sb in the roots of seedlings exposed to 2 *μ*M Sb(III) for 4 h. Deposition of Sb in the roots of two different rice cultivars was mainly found in the outer cell layers, mainly in the exodermal cells (Fig. 3, F and H). Low Sb was detected in the inner root tissues. However, in the *lsi1* mutants described below, much weaker signal of Sb was detected in the roots (Fig. 3, G and I).

### Identification of transporters for Sb uptake

**Figure 4.**
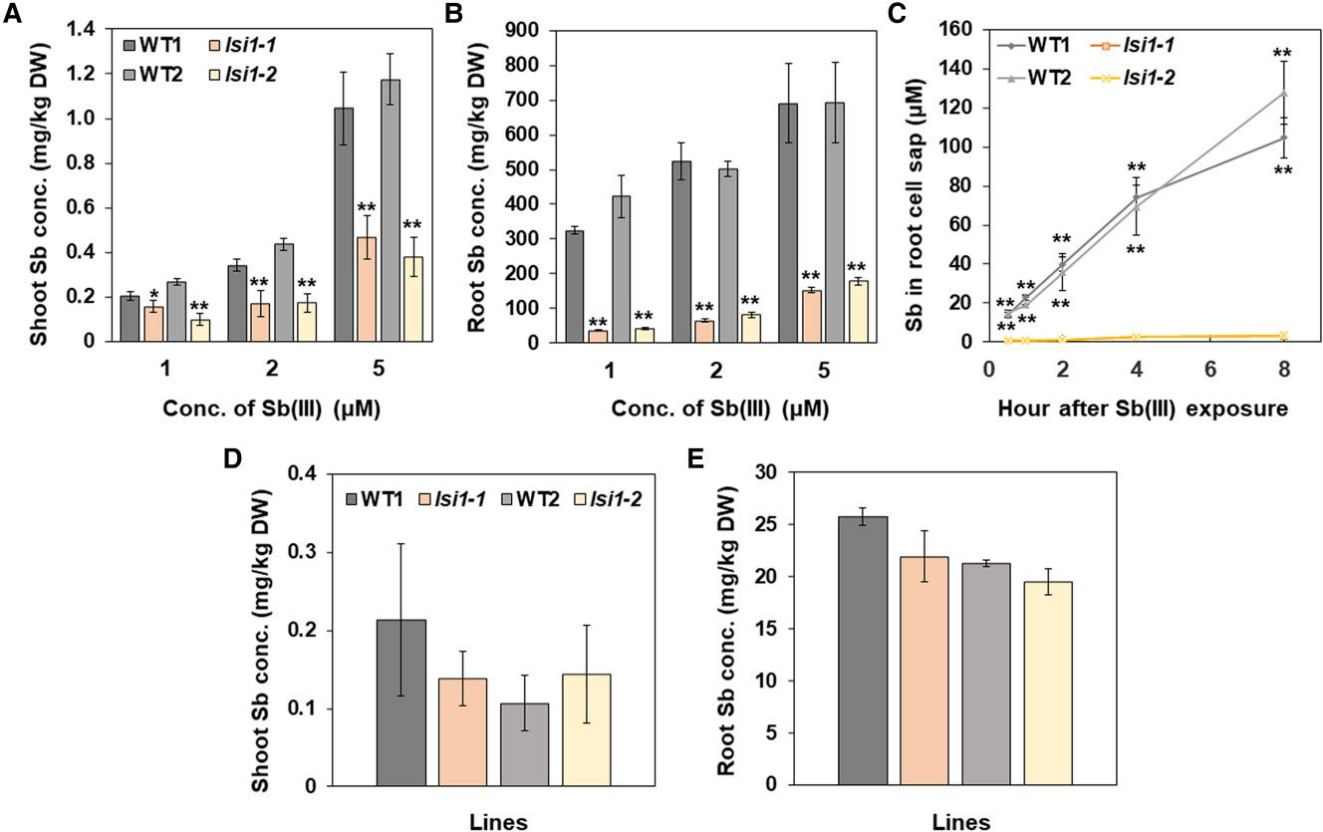
Effect of defect of Lsi1 on Sb accumulation in rice. **A** to **B)** Dose-dependent Sb concentration in the shoots (A) and roots (B). Seedlings (24-d-old) of two independent mutants (*lsi1-1* and *lsi1-2*) and their WTs [Nipponbare (WT1) and Oochikara (WT2)] were exposed to different concentration of Sb(III) including 1, 2, or 5 *μ*M for 1 d. **C)** Time-dependent Sb concentration in the root cell sap. The root cell sap was collected from seedlings (22-d-old) of two independent *lsi1* mutants and their WTs exposed to 2 *μ*M Sb(III) for 0.5, 1, 2, 4, and 8 h, respectively. **D** to **E)** Sb concentration in the shoots (D) and roots (E) of *lsi1* mutants and their WTs after exposure to 2 *μ*M Sb(V) for 1 d. The concentration of Sb in plant tissues and root cell sap was determined by ICP-MS. Data are means ± Sd of three to four biological replicates. Significant differences between *lsi1* mutants and their corresponding WTs are marked with *P < 0.05; **P < 0.01, by Student's *t*-test. Conc., concentration; DW, dry weight.

Since Sb(III) shows similar properties as As(III) in terms of size and structure (Pommerrenig et al. 2015), it was proposed that Sb(III) shares the same transporter for As(III). In fact, transporters identified in *E*. *coli* (GlpF), and yeast (Fps1p) transport both Sb(III) and As(III) (Sanders et al. 1997; Wysocki et al. 2001; Meng et al. 2004). AtNIP1; 1 in Arabidopsis also transports both Sb(III) and As(III) (Kamiya and Fujiwara 2009; Kamiya et al. 2009). All these transporters belong to aquaporin family. Since As(III) uptake is mainly mediated by Lsi1, a silicon transporter in rice (Ma et al. 2008), which showed transport activity for Sb(III) when expressed in the yeast (Bienert et al. 2008), we first investigated whether Lsi1 also functions as a major transporter for Sb(III) in rice. For this purpose, we used two independent *lsi1* mutants and compared their Sb accumulation with their WTs in a dose-dependent manner. At all Sb(III) supply concentrations tested, the mutants always showed much lower Sb concentration in both the roots and shoots compared with their WTs (Fig. 4, A and B). Especially, in the roots, the Sb concentrations in the mutants were only 10% to 26% of the WTs (Fig. 4B). Consistent with this result, much less Sb was accumulated in the root exodermis compared with the WTs revealed by LA-ICP-MS (Fig. 3, F to I).

To further estimate real Sb(III) uptake, we compared Sb concentration in the root cell sap between mutants and WTs. A time-course experiment showed that the Sb in the root cell sap increased with increasing exposure time in the WTs, but not in the mutants (Fig. 4C). At 8 h after the exposure, the Sb concentrations in the root cells were 33 to 35 times higher in the WTs than in the mutants (Fig. 4C).

We also examined the Sb accumulation in the WTs and *lsi1* mutants when they were exposed to Sb(V). In contrast to Sb(III), no difference in the Sb concentration of both the roots and shoots was found between mutants and WTs (Fig. 4, D and E). These results indicate that Lsi1 transports Sb(III), but not Sb(V).

**Figure 5.**
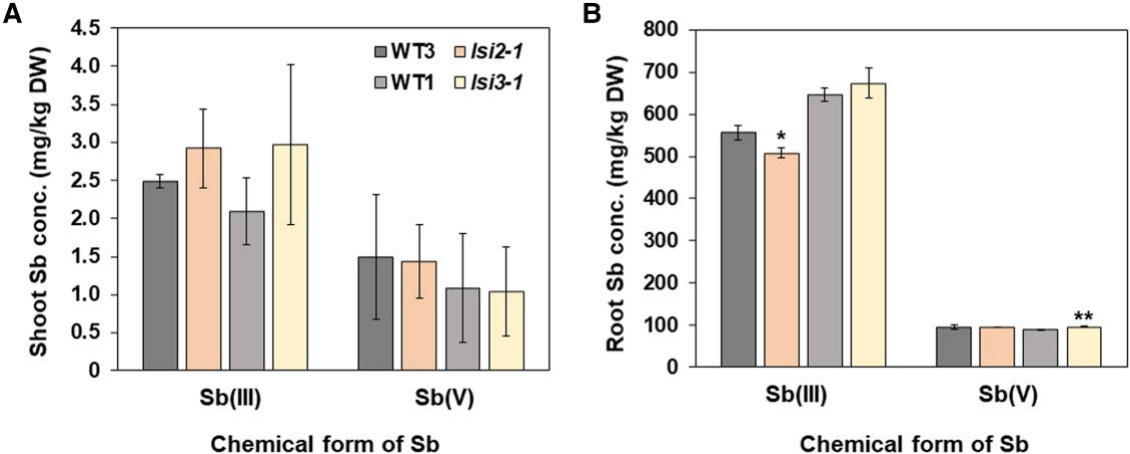
Effect of knockout of *Lsi2* and *Lsi3* on Sb accumulation in rice. 24-d-old seedlings of mutants (*lsi2-1*, *lsi3-1*) and their WTs [T-65 (WT3) and Nipponbare (WT1)] were exposed to 10 *μ*M Sb(III) or Sb(V) for 1 d. The concentration of Sb in shoots **A)** and roots **B)** was determined by ICP-MS. Data are means ± Sd of three biological replicates. Significant differences between *lsi2*, *lsi3* mutant and their corresponding WTs are marked with *P < 0.05; **P < 0.01, by Student's *t*-test. Conc., concentration; DW, dry weight.

Lsi2, an efflux transporter for Si is also involved in As(III) uptake (Ma et al. 2008). We then tested whether Lsi2 is also involved in Sb(III) uptake. However, in contrast to Lsi1, knockout of *Lsi2* did not affect the Sb accumulation in both the roots and shoots (Fig. 5, A and B). In addition, we also investigated the involvement of Lsi3 in Sb accumulation. Lsi3 is a homolog of Lsi2, and was reported to function in the xylem loading of Si although its involvement in As loading was not investigated (Huang et al. 2022). Similar to Lsi2, knockout of *Lsi3* did not affect the Sb accumulation in the roots and shoots (Fig. 5, A and B). These results indicate that Lsi2 and Lsi3 are not involved in Sb uptake and xylem loading.

### Transport activity of Lsi1 for Sb(III) and Sb(V) in yeast

**Figure 6.**
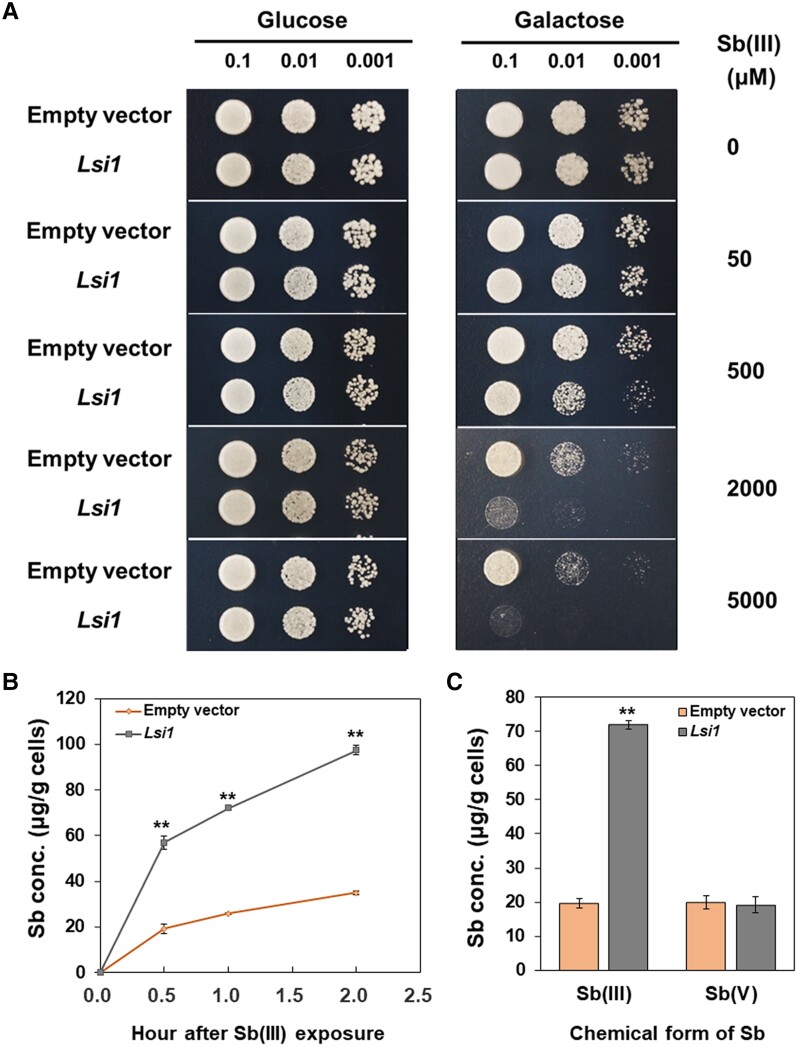
Transport activity of Lsi1 for Sb(III) and Sb(V) in yeast cells. **A)** Growth of yeast strain expressing an empty vector or *Lsi1* in a SD-U solid medium containing different concentration of Sb(III) in the presence of glucose or galactose. The plates were photographed after 48 h incubation. **B)** Sb concentration in the yeast cells expressing an empty vector or *Lsi1*. The yeast were exposed to 50 *μ*M Sb(III) for different time periods in the presence of galactose. **C)** Transport activity for Sb(V). Yeast cells expressing an empty vector or *Lsi1* were exposed to 50 *μ*M Sb(III) or Sb(V) for 1 h in the presence of galactose. The concentration of Sb in yeast cells was determined by ICP-MS after digestion. Data are means ± Sd of three biological replicates. Significant differences between empty vector and *Lsi1* are marked with **P < 0.01, by Student's *t*-test. Conc., concentration.

Lsi1 (OsNIP2; 1) was reported to transport Sb(III) in yeast although the results were not shown (Bienert et al. 2008). To confirm this result and further characterize Sb transport by Lsi1, we expressed *Lsi1* in WT yeast cells (INVSc-1) under the control of the Gal-inducible promoter. In the presence of glucose (no gene expression) as a control, there was no growth difference between yeast expressing *Lsi1* and empty vector (Fig. 6A), although the growth was gradually inhibited with increasing Sb(III) concentrations in the media. However, in the presence of galactose (expression of *Lsi1* was induced), the growth of yeast expressing *Lsi1* was inhibited more by Sb(III), especially at higher concentrations (> 500 *μ*M) compared with those expressing empty vector (Fig. 6A).

We then quantitated Sb in the yeast. A time-course experiment showed that yeast cells expressing *Lsi1* accumulated much higher Sb compared with the empty vector control (Fig. 6B).

We also examined whether Lsi1 is also able to transport Sb(V). Different from Sb(III), the yeast expressing *Lsi1* and empty vector did not show difference in Sb accumulation (Fig. 6C). Consistent with the result in rice (Fig. 4, D and E), these results indicate that Lsi1 is not able to transport Sb(V).

### Effect of Si addition on Sb accumulation

Above results clearly show that Sb(III) uptake is mediated by Lsi1, a Si permeable transporter (Figs. 4 and 6). We then investigated the effect of Si on Sb accumulation. The results showed that the addition of Si significantly reduced the Sb accumulation in both the roots and shoots (Supplementary Fig. S1), supporting our conclusion.

### Test of Sb toxicity in *lsi1* mutants

**Figure 7.**
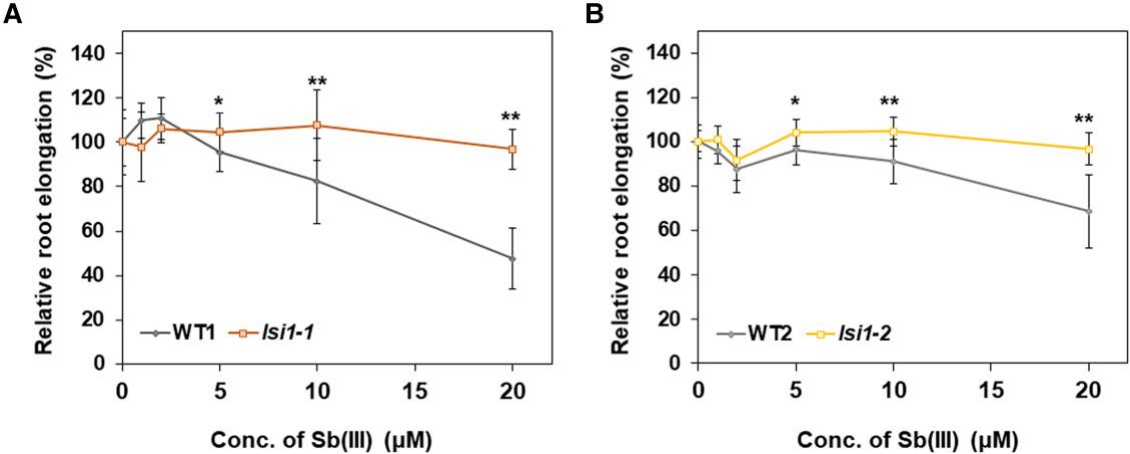
Effect of Sb(III) on root elongation of rice *lsi1* mutants and their wild types (WTs) rice. **A** to **B)** Relative root elongation. Seedlings (4-d-old) of two *lsi1* mutants [*lsi1-1* (A) and *lsi1-2* (B)] and their WTs (WT1 and WT2) were exposed to a solution (pH 5.6) containing 0, 1, 2, 5, 10, and 20 *μ*M Sb(III) for 24 h. The root length was measured before and after the treatment. Root elongation relative to no Sb treatment is shown. Data are means ± Sd of 9 to 10 biological replicates. Significant differences between *lsi1* mutants and their corresponding WTs are marked with *P < 0.05; **P < 0.01, by Student's *t*-test. Conc., concentration.

Sb shows toxicity at high concentrations, which inhibits root growth (Lu et al. 2023). Since *lsi1* mutants accumulated much less Sb in the roots, we examined whether this is associated with Sb toxicity. We exposed the plants to different concentrations of Sb(III) and monitored root elongation during 24 h. The root growth of both WTs and mutants was hardly inhibited at low Sb(III) concentrations (1 to 2 *μ*M) (Fig. 7, A and B). However, at higher Sb(III) concentrations (5 to 20 *μ*M), the root elongation was inhibited more in the WTs than in the mutants. These results indicate that knockout of *Lsi1* increased tolerance to Sb(III) due to decreased Sb uptake (Figs. 4 and 6).

## Discussion

In the present study, through detailed physiological characterization of Sb accumulation in rice at realistic Sb concentrations, we found that Sb(III) is the preferential form for the uptake (Fig. 1). This result is consistent with previous studies (Huang et al. 2012a; Ren et al. 2014). Since rice is cultivated under flooded conditions during most growth period, where Sb(III) is the predominant form, the contribution for Sb accumulation from Sb(III) is larger than that from Sb(V) (Fig. 1). Furthermore, speciation analysis showed that Sb is present in the form of Sb(III) in the root cells after uptake (Fig. 3B), suggesting that oxidation of Sb(III) did not occur in our experiment condition. We also found that the translocation of Sb from the roots to the shoots was very low (Fig. 1C). This is supported by high Sb concentration in the root cell sap, but a very low level of Sb in the xylem sap (Fig. 3, D and E), indicating that only a small part of Sb taken up was translocated to the shoots. Most Sb was deposited at the root outer cell layers, mainly in the exodermis (Fig. 3, F and H). This low translocation could be attributed to lack of efflux transporters for Sb in rice roots as discussed below.

Rice roots have a distinct anatomy, which is characterized by two Casparian strips at the exodermis and endodermis, and the formation of aerenchym (Enstone et al. 2002). Therefore, to transport an element from soil solution to the stele for subsequent translocation to the shoot, both influx and efflux transporters localized at both the exodermis and endodermis are required. The cooperation of influx–efflux transporters forms an efficient system for the uptake of mineral elements. This system has been well documented in rice for Si, Mn, and B (Ma and Yamaji 2008; Che et al. 2018; Huang et al. 2020, 2024). For example, Lsi1 functions as an influx transporter for Si, which is polarly localized at the distal side, while Lsi2 functions as an efflux Si transporter polarly localized at the proximal side (Ma et al. 2006, 2007). In this present study, we found that Lsi1 also transports Sb(III), but Lsi2 does not based on yeast assay and rice mutant analysis (Figs. 4 to 6). Therefore, once Sb(III) is taken up by Lsi1 localized at the exodermis, Sb was retained in this cell layer due to a lack of efflux Sb transporter for releasing Sb(III) toward the stele, resulting in heavy Sb deposition in the exodermis (Fig. 3, F and H). Surprisingly, the Sb concentration in root cell sap is very high (> 200 *μ*M) (Fig. 2A), when nontoxic Sb(III) (2 *μ*M) was added (Fig. 7). This suggests that Sb taken up by Lsi1 is sequestered into the vacuoles in the exodermis although the responsible transporter is unknown.

Lsi1 is initially identified as a channel-type transporter for silicic acid (Ma et al. 2006). Subsequent studies showed that Lsi1 is also permeable to arsenite (Ma et al. 2008) and selenite (Zhao et al. 2010). In the present study, we clearly show that Lsi1 is a major transporter for Sb(III). This is supported by that yeast expressing *Lsi1* showed transport activity for Sb(III) (Fig. 6), and that knockout of *Lsi1* resulted in 74% to 90% reduction of Sb in the roots (Fig. 4B). Increased tolerance to Sb(III) in *lsi1* mutants is the result of decreased uptake (Figs. 7 and 4). These results indicate that silicic acid, As(III), Se(IV), and Sb(III) share the same transporter for the uptake in rice roots. This is reasonable because their molecules are all present in the noncharged form and have similar sizes. Sb(III) has a molar volume of 62 cm^3^/mol, which is quite similar to that of As(III) (59 cm^3^/mol), silicic acid (54 cm^3^/mol), and Se(IV) (74 cm^3^/mol) (Pommerrenig et al. 2015).

Lsi1 belongs to Nodulin 26-like Intrinsic Protein (NIP) subfamily of aquaporin (Yamaji and Ma 2021). NIP is a unique subfamily of aquaporin that is present only in plants and is characterized by transporting metalloids. It was proposed that the Asn-Pro-Ala (NPA) and aromatic/arginine (ar/R) regions of aquaporins largely affect selectivity of substrates. Especially, the ar/R selectivity filter consisting of four dispersed amino acid residues represents the narrowest part of the channel pore and forming a size exclusion barrier that confers selectivity to particular substrates. Based on the sequence similarity and the ar/R selectivity filter, NIP subfamily can be subdivided into three subgroups, NIP I-III (Mitani et al. 2008). Typically, the filter is WVAR for NIP I, AIGR for NIP II, and GSGR for NIP III (Mitani et al. 2011; Pommerrenig et al. 2015; Yamaji and Ma 2021). They show different specificities for transport substrates. For example, NIP I is permeable to As(III) and Sb(III) (Bienert et al. 2008; Kamiya and Fujiwara 2009; Kamiya et al. 2009), while NIP II subgroup is permeable to both As(III) and boric acid (B(OH)_3_), but not to Si(OH)_4_. In contrast, NIP III subgroup is permeable to As(III), B(OH)_3_, and Si(OH)_4_ (Pommerrenig et al. 2015; Yamaji and Ma 2021). So far, only two NIP members; AtNIP1; 1 belonging to NIP I subgroup in Arabidopsis and Lsi1 (NIP2; 1) belonging to NIP III subgroup in rice in this study, was experimentally demonstrated to transport Sb(III) in planta although some other members also show transport activity for Sb(III) in yeast (Bienert et al. 2008; Kamiya and Fujiwara 2009). Interestingly, AtNIP1; 2 and AtNIP5; 1 do not mediate Sb uptake although they have high similarity with AtNIP1; 1 (Kamiya and Fujiwara 2009). It remains to be investigated whether other NIP members are involved in Sb(III) uptake or other transport processes in rice.

Lsi2 is responsible for releasing Si(OH)_4_ and As(III) (Ma et al. 2007, 2008), but not Sb(III) (Fig. 5). Since Sb is also present in the form of Sb(III) in the root cells (Fig. 3B), it is unknown why Lsi2 is not able to transport Sb(III). Different from Lsi1, Lsi2 is a proton–antiporter, which may have stricter requirements for the structure. In fact, although the uptake of metalloids into the root cells is somewhat similarly mediated by NIP members as discussed above, the efflux of these metalloids from root cells is driven by different types of transporters without any similarity to each other (Yamaji and Ma 2021). For example, B efflux is mediated by the High B Requiring (BOR) family transporter (Yoshinari and Takano 2017), while Si efflux from root cells is mediated by Lsi2 transporter, which has no similarity with BOR. Given that most Sb taken up is retained in the root exodermis, it is unlikely that rice root has an efflux transporter for Sb(III).

Knockout of *Lsi1* resulted in significant reduction of Sb accumulation (Fig. 4). However, since this knockout also affects Si accumulation, which is important for high and stable rice production, it is not a wise way to utilize this mutant for low Sb accumulation in agriculture. There may be two ways to lower Sb accumulation in rice in the future. One is to manipulate selectivity of Lsi1 in order to block Sb rather than Si. Recently, the crystal structure of Lsi1 has been revealed (Saitoh et al. 2021). The transmembrane helical orientations of Lsi1 are different from other aquaporins, characterized by a unique, widely opened, and hydrophilic selectivity filter composed of five residues. Based on this structural information, selectivity for Sb and Si could be examined by manipulating key amino acid residues in the future. Another easy way may be to apply Si fertilizers to soil. Si addition significantly reduced Sb accumulation in the shoots (Supplementary Fig. S1). This antagonistic effect may be caused by competition for Lsi1 between Si and Sb.

In conclusion, we found that Lsi1 is a major transporter for Sb(III), a preferential form for rice uptake. Rice shows very low translocation rate of Sb from the roots to shoots due to lack of efflux transporter, resulting in deposition of Sb at the root exodermis.

## Materials and methods

### Plant materials and growth conditions

Following rice (*Oryza sativa*) mutants and their corresponding wild types (WTs) were used in this study: *lsi1-1* and *lsi1-2* defective in Si uptake and their WTs (WT1, Nipponbare; WT2, Oochikara), *lsi2-1* defective in Si uptake and its WT (WT3, T-65), *lsi3-1* defective in Si xylem loading and its WT (WT1, Nipponbare). These mutants were isolated or generated previously (Ma et al. 2002, 2006, 2007; Chiba et al. 2009; Huang et al. 2022). Seeds of WTs and mutants were soaked in water in the dark at 30 °C. The germinated seeds (2 d later) were transferred onto a plastic net floating on a 0.5 mM CaCl_2_ solution in a 1.2-L plastic pot. After 4 to 6 d, the seedlings were transferred to a 3.5-L plastic pot containing half-strength Kimura B solution (Ma et al. 2002). FeSO_4_ was freshly prepared and added at a final concentration of 2 *μ*M. The plants were grown in a controlled greenhouse at 25 °C to 30 °C with natural light. The nutrient solution was changed every 2 d. Seedlings were used in the following experiments. All experiments were performed at least twice each with independent replicates as described in each figure legend.

### Physiological characterization of Sb accumulation

We investigated different form of Sb including Sb(III) or Sb(V) on Sb accumulation in a dose- and time-dependent manner. For a dose-dependent experiment, 24-d-old seedlings of Nipponbare (WT1) prepared as described above, were exposed to 1, 2, 5, or 10 *μ*M of Sb(III) or Sb(V) in the nutrient solution for 1 d. In a time-dependent experiment, the seedlings were exposed to 2 *μ*M Sb(III) or Sb(V) for 1, 3, and 7 d, respectively. Sb(III) and Sb(V) were respectively prepared using potassium antimonyl tartrate trihydrate (C_8_H_4_K_2_O_12_Sb_2_·3H_2_O) (Nacalai Tesque, Kyoto, Japan) and potassium hexahydroxoantimonate {KSb(OH)_6_} (Wako, Osaka, Japan). At harvest, the roots were washed with pre-cooled 5 mM CaCl_2_ solution three times, and then separated from the shoots. The concentration of Sb in the roots and shoots was determined as described below. Translocation was calculated as (content in the shoot/content in the whole plant × 100).

### Analysis of Sb partition in rice roots

To investigate the partition of Sb in rice roots, 24-d-old seedlings of Nipponbare were exposed to 2 *μ*M Sb(III) or Sb(V). After 1 d, root cell sap was collected and separated from the root cell wall. Briefly, the roots were washed with pre-cooled 5 mM CaCl_2_ solution three times, blotted and immediately placed on a filter in a tube and frozen in liquid nitrogen. After thawing at room temperature, the cell sap was collected by centrifugation at 15,000 rpm for 10 min and weighed. The fresh weight was recorded before and after centrifugation. The remaining part including the cell wall and part of cell sap was dried at 70 °C for at least 2 d and weighed again. The difference between root fresh weight before centrifugation and dry weight was calculated as the total cell sap volume. The remaining part was subjected to digestion. Sb in the root cell sap and the remaining part was determined as described below. The portion of Sb in the cell wall was calculated based on the weight difference and Sb concentration in the cell sap and the remaining part.

### Sb speciation analysis by HPLC-ICP-MS

To analyze Sb speciation within the rice root after uptake, seedlings (cv. Nipponbare, 22-d-old) were exposed to a nutrient solution containing 2 *μ*M Sb(III) or Sb(V) for 4 h. The root cell sap was collected as described above. The samples were immediately subjected to analysis by high performance liquid chromatography (HPLC)-inductively coupled plasma-mass spectrometry (ICP-MS) for speciation analysis with a column (G3288-80000, Agilent). Citric acid at 100 mM was used as mobile phase with a pH of 4.5, which was adjusted with 28% (w/w) ammonia solution (Hansen et al. 2011). Peak heights were used for quantification analysis of Sb(III) and Sb(V) by using standard curves run under the same conditions.

### Collection of xylem sap

To collect xylem sap, seedlings (22-d-old, cv. Nipponbare) were exposed to a nutrient solution containing 2 *μ*M Sb(III) or Sb(V). After 4 h, shoots were excised with a razor from 2 cm above the root–shoot junction. After discarding the first drop, xylem sap was collected from the cut surface for 60 min by using a micropipette and subsequently subjected to Sb determination as described below.

### Sb deposition pattern analysis by LA-ICP-MS

To observe the distribution pattern of Sb at different tissues of the roots, 5-d-old seedlings of two *lsi1* mutants and their WTs were exposed to a 0.5 mM CaCl_2_ solution containing 2 *μ*M Sb(III). After 4 h, roots (20 mm from the root apex) of each line were sampled and subjected to Sb determination with laser ablation (LA) device (NWR213; New Wave Research) and ICP-MS (8900; Agilent Technologies) operated in helium mode. The sample preparation procedures and mapping method were the same as described previously (Yamaji and Ma 2019). Two biological replicates of each line were analyzed, which showed the similar results.

### Analysis of Sb accumulation in rice mutants

To investigate whether Lsi1 is involved in Sb uptake and accumulation in rice, a kinetic experiment was performed by exposing two independent mutants (*lsi1-1* and *lsi1-2*) and their WTs (22-d-old) to a nutrient solution containing 1, 2 or 5 *μ*M Sb(III). After 1 d, the roots were washed with pre-cooled 5 mM CaCl_2_ solution three times and separated from the shoots.

To further characterize Sb uptake, we compared Sb concentration in the root cell sap between *lsi1* mutants and WTs in a time-dependent manner. After the seedlings (24-d-old) were exposed to a nutrient solution containing 2 *μ*M Sb(III) for 0.5, 1, 2, 4, and 8 h. The roots were sampled and used for root cell sap collection as described above.

To test whether Lsi1 is involved in Sb(V) uptake and accumulation in rice, seedlings (22-d-old) were exposed to a nutrient solution containing 2 *μ*M Sb(V) for 1 d. Plants were harvested as mentioned above.

Similar experiments were conducted using *lsi2* and *lsi3* mutants. 24-d-old seedlings were exposed to a nutrient solution containing 10 *μ*M Sb(III) for 1 d. Plants were sampled as described above.

### Effect of Si on Sb accumulation

To investigate the effect of Si addition on Sb accumulation, seedlings (14-d-old, cv. Nipponbare) were exposed to a nutrient solution containing 2 *μ*M Sb(III) in the presence or the absence of 1 mM Si as silicic acid. Silicic acid was prepared as previously reported (Ma et al. 2002). The solution was changed every 2 d. After 14 d, the roots and shoots were harvested as described above and subjected to determination of Sb by ICP-MS as described below.

### Sb transport activity assay in yeast

To test the transport activity of Lsi1 for Sb(III) and Sb(V) in yeast, we used yeast strain INVSc-1 (S.c. easy comp transformation kit; Invitrogen) expressing *Lsi1* or empty vector under the control of galactose-inducible promoter generated in a previous study (Zhao et al. 2010). The transformed yeasts were grown on synthetic SD-U medium containing 0.67% (w/v) yeast nitrogen base without amino acids (Difco), 0.19% (w/v) mixed amino acid without uracil, 0.003% (w/v) adenine hemisulfate, 2% (w/v) glucose, and 2% (w/v) agar at pH 5.8 for selection.

To examine the effect of Sb(III) exposure on yeast growth, yeast expressing *Lsi1,* or empty vector was precultured in SD-U liquid medium and then washed with sterilized Milli-Q water for three times. The cell suspension was serially diluted at OD_600_ of 0.1, 0.01, and 0.001, and 5 *μ*l of each dilution was spotted on SD-U solid medium containing different concentration of Sb(III) in the presence of glucose (as a control) or galactose. Pictures of yeast growth were taken at 48 h after incubation at 30 °C.

To determine Sb uptake, yeast cells expressing *Lsi1* or empty vector were precultured in SD-U liquid medium containing 2% (w/v) galactose until OD_600_ of 1.5 to 2.0. Subsequently, the cells were collected by centrifugation and re-suspended in SD-U liquid medium with galactose containing 50 *μ*M Sb(III) and incubated at 30 °C with gently shaking. At different time points (0, 0.5, 1, 2 h), yeast cells were sampled and collected by centrifugation. After washed three times with pre-cooled 5 mM CaCl_2_ solution, the yeast cells were dried and subjected to digestion and Sb determination as described below. To compare the transport activity for Sb(III) and Sb(V), the yeast cells were exposed to a solution containing 50 *μ*M Sb(III) or Sb(V). After 1-h incubation, the yeast cells were harvested as described above.

### Effect of Sb(III) on relative root elongation

To evaluate the effect of Sb(III) on root elongation, 4-d-old seedlings of two independent *lsi1* mutants and their WTs were exposed to 0.5 mM CaCl_2_ solution containing 0, 1, 2, 5, 10, or 20 *μ*M Sb(III) (pH 5.6) for 24 h. The root length was measured with a ruler before and after the treatment. Relative root elongation was calculated as [root elongation with Sb(III) treatment]/[root elongation without Sb(III)] × 100.

### Elements determination in root cell sap, xylem sap, plants, and yeast cells

Plant samples harvested were dried at 70 °C for at least 2 d, and then digested by 61% HNO_3_ (w/v) as described previously (Gu et al. 2022). Yeast cells were dried at 70 °C overnight before digested by 61% HNO_3_ (w/v) at temperatures of 90 °C for 1 h and 110 °C for 10 min. The concentration of Sb in the digestion solution and root cell sap as well as xylem sap were determined with ICP-MS (7700X and 8900; Agilent Technologies).

### Statistical analysis

Statistical comparison was performed by Student's *t*-test. The significance of differences was defined as: *P < 0.05; **P < 0.01.

### Accession numbers

Sequence data from this article can be found in the GenBank/EMBL data libraries under accession numbers AB222272 (*Lsi1*), AB222273 (*Lsi2*), and LC069370 (*Lsi3*).

## Supplementary Material

kiae289_Supplementary_Data

## Supplementary data

The following materials are available in the online version of this article.

**Supplementary Figure S1.** Effect of Si addition on Sb accumulation in rice.

## Dive Curated Terms

The following phenotypic, genotypic, and functional terms are of significance to the work described in this paper:

antimony CHEBI: CHEBI:30304

citric acid CHEBI: CHEBI:30769

root AmiGo: PO:0009005

silicic acid CHEBI: CHEBI:26675
