# Supplementary material for: Tissue-specific deposition, speciation and transport of antimony in rice
Source: Plant Physiol. 2024 May 18;195(4):2683–93. doi: 10.1093/plphys/kiae289 (PMC11288759; doi:10.1093/plphys/kiae289)
Supplement: kiae289_Supplementary_Data [file kiae289_supplementary_data.zip › PP2024RA00346R1_Supplemental_Material.pdf]

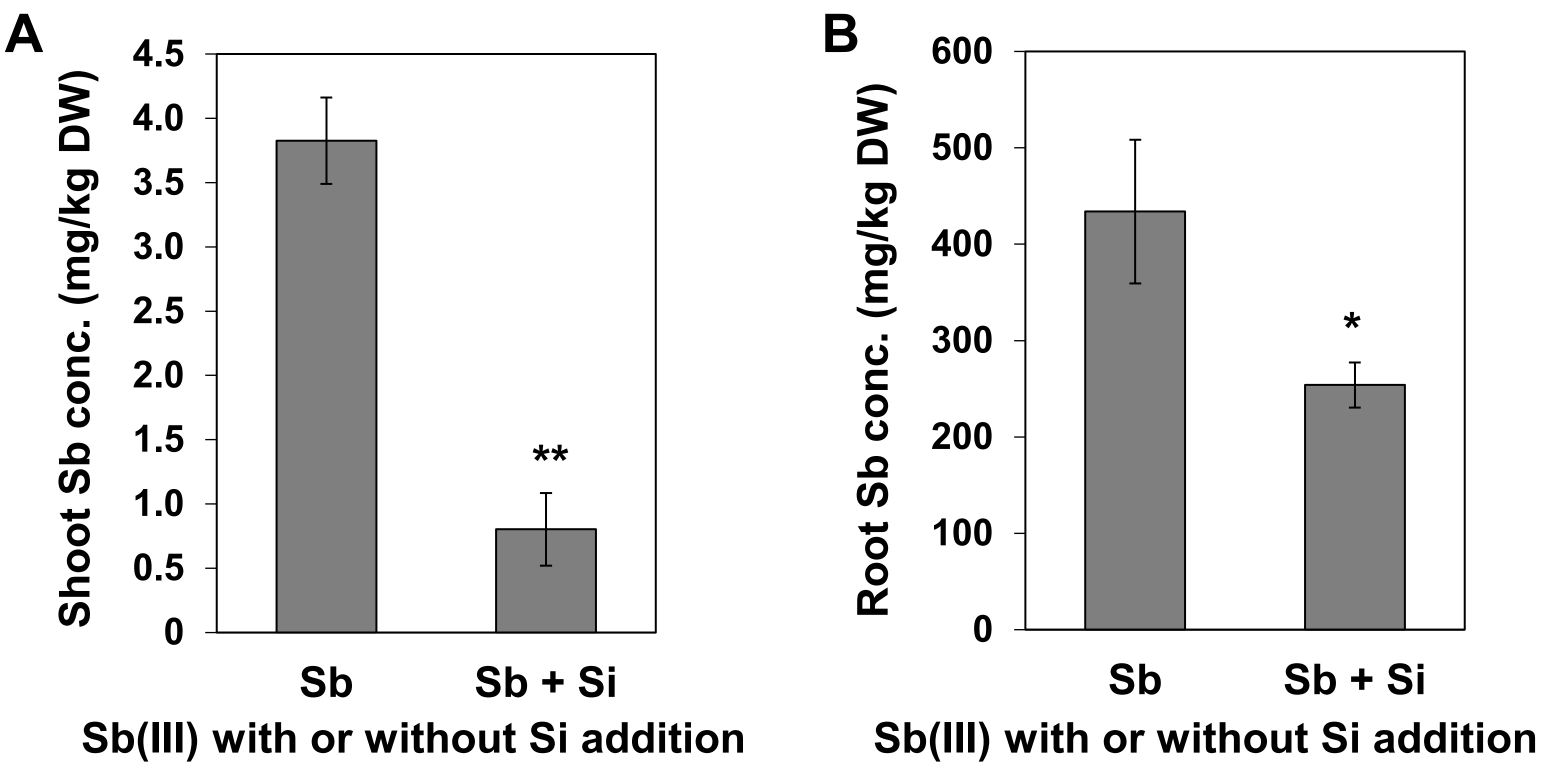

Supplementary Figure S1. Effect of Si addition on Sb accumulation in rice. 14-d-old seedlings (cv. Nipponbare) were exposed to a nutrient solution containing 2 μM Sb(III) with or without 1 mM Si (silicic acid) addition for 14 d. The concentration of Sb in shoots (A) and roots (B) was determined by ICP-MS. Data are means ± SD of 3 biological replicates. Significant differences between Sb and Sb + Si are marked with *, $P < 0.05$; **, $P < 0.01$, by Student's *t*-test. Conc., concentration; DW, dry weight.
