# Supplementary material for: Tissue-specific deposition, speciation and transport of antimony in rice
Source: Plant Physiol. 2024 May 18;195(4):2683–93. doi: 10.1093/plphys/kiae289 (PMC11288759; doi:10.1093/plphys/kiae289)
Supplement: kiae289_Supplementary_Data [file pp.00346.2024-s03.pdf › pp.00346.2024-s03.pdf]

Plant Physiology®

Tissue-specific deposition, speciation and transporter of antimony in rice

Hengliang Huang, Naoki Yamaji, and Jian Feng Ma

Review Timeline:

Submission Date:	04-Mar-2024
Editorial Decision:	12-Apr-2024
Revision Received:	18-Apr-2024
Accepted:	02-May-2024

12-Apr-2024

Prof. Jian Feng Ma
Okayama Daigaku
Institute of Plant Science and Resources
Kurashiki 710-0046
Japan

RE: Tissue-specific deposition, speciation and transporter of antimony in rice

Dear Dr. Jian Feng Ma:

We are pleased to accept your manuscript for publication in the Plant Physiology. This acceptance is contingent on revision based on the comments of our reviewers.

Please highlight all changes and include a detailed annotation to changes to the text, with line numbers, and noting your responses to the comments.

To submit your revised manuscript, click:

Link Not Available

If you cannot return the revised manuscript within 8 weeks of receipt, please let us know. Otherwise, we will assume that you have elected not to revise the manuscript and are withdrawing it.

Thank you for allowing us to review your work. We look forward to hearing from you soon.

Sincerely,

Editor, Plant Physiology

-- Reviewer comments:
Reviewer #1 (Comments for the Author):

Antimony is a toxic element for human. Rice is a staple food in a lot of countries and its contamination can cause health issues. However, its molecular form of uptake by rice and molecular mechanisms have been unclear. In this manuscript, the author identified 1) Sb(III) is the major form of Sb taken up by rice, 2) Lsi1, a silicon (Si) transporter, transports Sb(III), 3) Si supplementation reduce Sb(III) uptake and toxicity, using analytical chemistry and mutants. The conclusions are well supported by the results. I have only minor comments.

1. Add reference to Line 50, starting with "Furthermore, Sb...."

2. In line 94-98, add the value of Sb(III) concentration in soil in µM (Lintschinger et al., 1998; Hammel et al., 2000) to rationalize the concentration the author used in this manuscript.

3. For all fig, please describe the combination of statistical analysis (Student's t test). E.g. between WT and mutant; between empty vector and Lsi1

4. Please state which allele the author used for lsi2 and lsi3. E.g. lsi2-1 etc.

5. Collect the position of brackets in Fig. 3C (Sb(V)), Fig. 2A-C and 3D-E (Sb(III)).

6. "0" (zero) should not be 0.0, 0.00. It should be "0".

Reviewer #2 (Comments for the Author):

Authors show here that the Si-, As-, and Se- transporter Lsi1 (OsNIP2;1) is transporting Sb in planta. Similar to their prior papers each showing the Lsi1-mediated transport of either of the above metalloids, authors collect evidence for Lsi1-mediated Sb transport using yeast uptake tests and established lsi1-mutant lines. What is new here is the employment of the LA-ICP-MS

technique to visualize Sb deposition in root cell layers. The experiments were carried out using appropriate methods and results were presented in a comprehensible manner.
I am not so sure, however, about the physiological relevance of the Sb transport in rice and if it justifies a full-scale paper. While transport of metalloids Si and Se is physiologically relevant, and As toxicity is a known and severe problem for rice cultivation, a physiological role for Sb is questionable and toxic levels are hard to reach in natural occurring soils. Can the authors therefore estimate the relevance of Sb transport and toxicity under natural and agricultural conditions or give examples for occurrence of Sb toxicity in natural or agricultural habitats? Maybe the fact that antimony is in fact not a big problem for rice since it is in most cases outcompeted by Si, is the important message to convey.

Some specific points:

Results:
In the first paragraph authors should shortly describe the experimental conditions applied, e.g. how plants were grown and at which developmental stage SB was applied.

Figure 1, D, E: It is not quite clear to me what the statistical differences are referring. Is it the significant difference between Sb(III) and Sb(V) at each individual time point? Or the time-dependent uptake? If so, authors might consider using one-way ANOVA coupled with a post-hoc test instead to test for significance of time-dependent uptake instead.

Figure 3: LA-ICP-MS of Sb in WT and Lsi1 mutants: How many plants per line were examined and how big was the variance in Sb deposition?

Figure 4: Authors clearly show that accumulation of Sb(III) as the dominant Sb form in roots of lsi1 mutants is reduced and reaches only about one tenth of the content compared to WT (Fig 4B). Can the authors give any explanation why this ratio is not reflected in shoots (about 50% of WT levels for lsi1-1, and about 33% of WT levels for lsi1-2), when Sb translocation from roots to shoots is only passive?
Moreover, the bars and error bars showing Sb(III) contents after application of 2 µM Sb in Figures 4C and Fig 4D in shoot and root, respectively, look identical to the bars presented in Figures 4A and 4B. Same values should not be used for different figures. Please check.

We thank two reviewers for their positive and constructive comments! We have revised the manuscript according to the comments as detailed below.

Reviewer #1 (Comments for the Author):

Antimony is a toxic element for human. Rice is a staple food in a lot of countries and its contamination can cause health issues. However, its molecular form of uptake by rice and molecular mechanisms have been unclear. In this manuscript, the author identified 1) Sb(III) is the major form of Sb taken up by rice, 2) Lsi1, a silicon (Si) transporter, transports Sb(III), 3) Si supplementation reduce Sb(III) uptake and toxicity, using analytical chemistry and mutants. The conclusions are well supported by the results. I have only minor comments.

1. Add reference to Line 50, starting with "Furthermore, Sb...."

Response: We have added references as shown in line 59-60.

2. In line 94-98, add the value of Sb(III) concentration in soil in μM (Lintschinger et al., 1998; Hammel et al., 2000) to rationalize the concentration the author used in this manuscript.

Response: We have shown the value of Sb(III) concentration in the soil in μM (Line104-105).

3. For all fig, please describe the combination of statistical analysis (Student's t test). E.g. between WT and mutant; between empty vector and Lsi1

Response: We have added the description of the combination of statistical analysis in revised figure legends.

4. Please state which allele the author used for lsi2 and lsi3. E.g. lsi2-1 etc.

Response: We have given the information about allele used for *lsi2* and *lsi3* mutant in the revised manuscript and figure.

5. Collect the position of brackets in Fig. 3C (Sb(V)), Fig. 2A-C and 3D-E (Sb(III)).

Response: We have corrected the position. Thank you for your careful reading!

6. "0" (zero) should not be 0.0, 0.00. It should be "0".

Response: We have changed “0.0”, “0.00” to “0” in all figures.

Reviewer #2 (Comments for the Author):

Authors show here that the Si-, As-, and Se- transporter Lsi1 (OsNIP2;1) is transporting Sb in planta. Similar to their prior papers each showing the Lsi1-mediated transport of either of the above metalloids, authors collect evidence for Lsi1-mediated Sb transport using yeast uptake tests and established lsi1-mutant lines. What is new here is the employment of the LA-ICP-MS technique to visualize Sb deposition in root cell layers. The experiments were carried out using appropriate methods and results were presented in a comprehensible manner.

I am not so sure, however, about the physiological relevance of the Sb transport in rice and if it justifies a full-scale paper. While transport of metalloids Si and Se is physiologically relevant, and As toxicity is a known and severe problem for rice cultivation, a physiological role for Sb is questionable and toxic levels are hard to reach in natural occurring soils. Can the authors therefore estimate the relevance of Sb transport and toxicity under natural and agricultural conditions or give examples for occurrence of Sb toxicity in natural or agricultural habitats? Maybe the fact that antimony is in fact not a big problem for rice since it is in most cases outcompeted by Si, is the important message to convey.

Response: Thank you for your concern! I agree with you that Sb contamination is not as serious as As, but it does a problem in some areas due to anthropogenic activities such as mining, smelting, shooting, and burning of fossil fuels. For example, it was reported that Sb concentration in soil was as high as 278.2-5045 mg/kg at an antimony mining and smelting area in Hunan, China, where rice is cultivated. We have added some background on Sb contamination in the Introduction of the revised manuscript.

.

Some specific points:

Results:

In the first paragraph authors should shortly describe the experimental conditions applied, e.g. how plants were grown and at which developmental stage SB was applied.

Response: We have added some brief description of the experimental conditions in the first paragraph as indicated.

Figure 1, D, E: It is not quite clear to me what the statistical differences are referring. Is it the significant difference between Sb(III) and Sb(V) at each

individual time point? Or the time-dependent uptake? If so, authors might consider using one-way ANOVA coupled with a post-hoc test instead to test for significance of time-dependent uptake instead.

Response: We compared Sb(III) and Sb(V) at different time points and found that there was a significant difference between two forms. We have added the information on the statistics.

Figure 3: LA-ICP-MS of Sb in WT and Lsi1 mutants: How many plants per line were examined and how big was the variance in Sb deposition?

Response: We used two plants of each line and obtained similar results as shown below. We have added some description on this point in the revised manuscript.

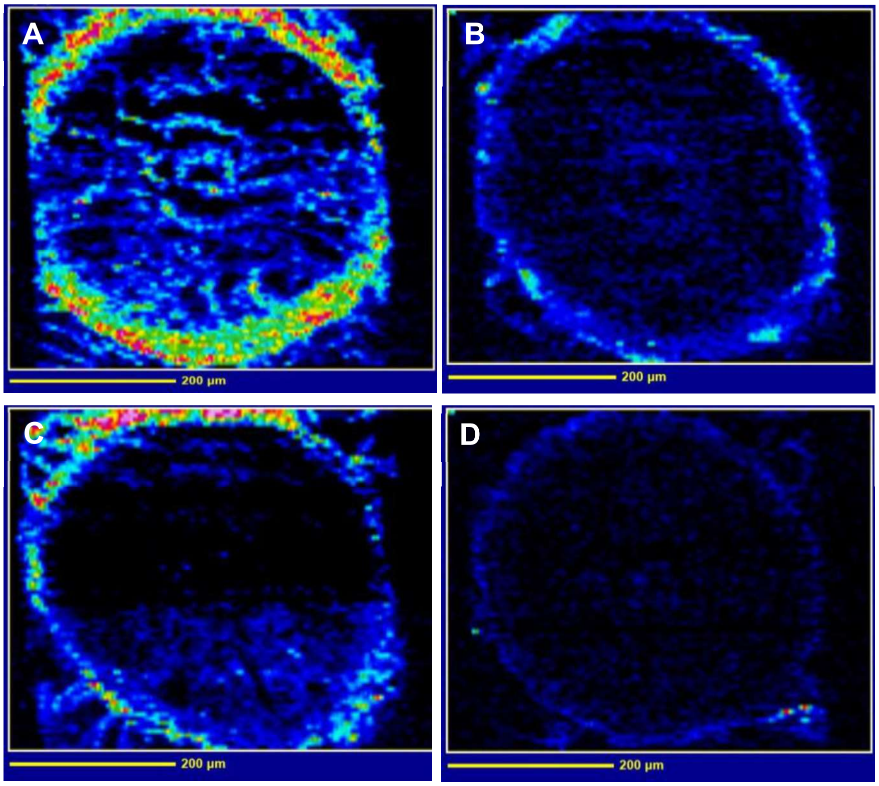

Figure 4: Authors clearly show that accumulation of Sb(III) as the dominant Sb form in roots of lsi1 mutants is reduced and reaches only about one tenth of the content compared to WT (Fig 4B). Can the authors give any explanation why this ratio is not reflected in shoots (about 50% of WT levels for lsi1-1, and about 33% of WT levels for lsi1-2), when Sb translocation from roots to shoots is only passive?

Response: To transport a mineral element from soil to the shoots, several transporters including uptake, vacuolar sequestration and xylem loading at least are required. Lsi1 is involved in the first step of Sb uptake, so its knockout significantly decreased root Sb accumulation. However, as we discussed in the paper, due to lack of efflux transporters such as Lsi2 and Lsi3, most Sb was accumulated at the exodermis, resulting in small difference in the shoot Sb accumulation between WT and mutant.

Moreover, the bars and error bars showing Sb(III) contents after application of 2 μM Sb in Figures 4C and Fig 4D in shoot and root, respectively, look identical to the bars presented in Figures 4A and 4B. Same values should not be used for different figures. Please check.

Response: Thank you for your comments! We have deleted this part in the revised Figure 4.

02-May-2024
Prof. Jian Feng Ma
Okayama Daigaku
Institute of Plant Science and Resources
Kurashiki 710-0046
Japan

MSID: PP2024-RA-00346R1
MS TITLE: Tissue-specific deposition, speciation and transporter of antimony in rice

Dear Dr. Jian Feng Ma:

I am pleased to inform you that your revised manuscript "Tissue-specific deposition, speciation and transporter of antimony in rice" has been accepted for publication in Plant Physiology. Your manuscript will be checked for consistency with journal formatting and image standards by one of our Science Editors, who will contact you shortly. They may have a number of queries and suggestions for you. Please keep in mind that they work to increase the online visibility of your article and its attraction for readers from the widest of backgrounds. You will have the opportunity to review any revisions, and I hope you will work constructively with them to get the most out of your research. You will also have the opportunity to review all changes when you receive the author proofs for your article.

Once validated for publication, your article will be sent to a Science Editor for a final review of your manuscript and source files. After your final files are uploaded, your article will appear in the next available issue and the online Preview version of your article will be posted after your signed Copyright and Checklist forms and final source files are received.

Please be sure to include the following in your manuscript file:
Harvard style citations
List of all author contributions and funding information at the beginning of manuscript after the titles and author list.
List all supplemental materials associated with the article, with titles, at the end of the ms, before the references and acknowledgments section.
Complete Figure Legends for all figures appearing in the article listed before the Literature Cited.

Thank you and congratulations on your Plant Physiology paper!

Sincerely,
Editor, Plant Physiology

=====
FIRST AUTHOR PROFILES
=====

We recognize the hard work that goes into being First Author of a Plant Physiology paper by publishing a "First Author Feature" for each First Author alongside your paper.

If the first author(s) would like to submit information for a feature on Facebook and Twitter, fill out the form by clicking on the link below:

Plant Physiology First Author Feature Form: https://forms.office.com/r/ZBCGZfhm8F
If you cannot access the form through the link above, please contact Rachel at rbelsky@aspb.org.

To upload an image of yourself to accompany your profile, please put your photo in the correct journal folder at the link below. You must label your image: Author First Name Last Name Article Title.

First Author Profile Image Dropbox: https://www.dropbox.com/scl/fo/xz029p7qjr2jml2rp2kmq/h?dl=0&rlkey=6fkxcaqmxx35ap152b2osy1fz

=====

IMPORTANT REMINDER: PEER REVIEW REPORTS
=====

If you opted to publish a peer review report along with your article during the original submission process, it will be prepared by the editorial staff and publicly posted with your manuscript, inside the zip file that contains any other supplemental material. As a reminder, the peer review report is a public record of all comments from editors and reviewers, as well as your prior responses, as you received them in the decision letters for each draft of your manuscript. If you agreed to publish this report and have changed your mind, or are not sure if you selected this option, please contact the editorial office as soon as possible before signing the license agreement from our publisher.

-- Reviewer comments:
Reviewer #1 (Comments for the Author):

All of my comments have been well addressed. Please note that the position of the blancket is still strange in Figure 2 A to C (Sb(III), Fig. 3D and E Sb(III), but it might be due to the difference of OS or software version.

Reviewer #2 (Comments for the Author):

The authors have adressed all of my concerns. I have no further issues and recommend accepting the manuscript for publication.
